# Are cortical microvascular raspberries caused by cerebral hypoperfusion? An exploratory pathological study

**DOI:** 10.1016/j.cccb.2021.100026

**Published:** 2021-08-17

**Authors:** Henric Ek Olofsson, Mattias Haglund, Elisabet Englund

**Affiliations:** Division of Pathology, Department of Clinical Sciences Lund, Lund University, Sölvegatan 25B, 22185 Lund, Sweden

**Keywords:** Cerebral angiogenesis, Cerebral hypoperfusion, Cerebral ischemia, Cerebral neovascularization, Cerebrovascular disease

## Abstract

**Introduction:**

This retrospective study investigated a cortical microvascular formation, termed a ‘raspberry’ due to its appearance under a bright-field microscope. We examined whether there is support for the hypothesis that raspberry formation is an angiogenic process induced by cerebral hypoperfusion.

**Materials and Methods:**

Raspberries were manually quantified in haematoxylin and eosin-stained cortical sections from the anterior frontal lobe of deceased individuals who had undergone a diagnostic neuropathological examination at the Department of Pathology, Lund, Sweden, during April 2019–January 2021. Subjects represented consecutively received cases during this 22-month period. The raspberry density was compared between subjects according to variables collected from medical records and autopsy reports: age, sex, hypertension, diabetes mellitus, atrial fibrillation, orthostatic hypotension, chronic heart failure, acute circulatory failure, aortic atherosclerosis, atherosclerosis of the basal cerebral arteries (referred to as ‘cerebral atherosclerosis’), cerebral small vessel disease, cerebral amyloid angiopathy, cerebral infarction, and ischaemic white matter disease.

**Results:**

62 subjects were included. The mean age was 71.9 years (range 46–97 years). 21 subjects (33.9%) were female. Independent-samples *t*-test showed a higher raspberry density in subjects with cerebral atherosclerosis (*p* = 0.029; 95% CI 0.7, 11.6 raspberries/cm²). The higher raspberry density in subjects with cerebral atherosclerosis remained in multiple linear regression (*p =* 0.003; 95% CI 2.3, 11.1 raspberries/cm²).

**Conclusion:**

This exploratory study indicates that cortical raspberries could be associated with cerebral atherosclerosis. The remaining results were inconclusive but motivate further examination of variables such as acute circulatory failure.

## Highlights

• An exploratory study on the ‘raspberry’, a type of cortical microvascular formation.
• Hypothesis: raspberries form by angiogenesis induced by cerebral hypoperfusion.
• Are raspberries associated with clinical or pathological markers of hypoperfusion?
• Data: histopathological raspberry quantification, medical records, autopsy reports.
• Raspberries were associated with atherosclerosis of the basal cerebral arteries.

## 1 Introduction

Ischaemic lesions of the brain can be caused by occlusion of blood vessels or by hypoperfusion. Suggested mechanisms of cerebral hypoperfusion include systemic hypotension, cerebrovascular disease, or a combination thereof. Acute hypoperfusion during cardiovascular surgery [1, 2], cardiac arrest [3], or other causes of circulatory shock [4] can result in both cortical and subcortical ischaemic lesions, while chronic or recurrent hypoperfusion has been suggested as a possible mechanism of ischaemic white matter disease [5]. In the latter case, underlying causes are believed to include cerebral small vessel disease and cerebral amyloid angiopathy [5, 6]. Further, atherosclerosis of larger cerebral blood vessels [7], orthostatic hypotension [8], chronic heart failure [9, 10], and atrial fibrillation [11] have been associated with reduced cerebral blood flow, possibly indicating a link to hypoperfusion. For orthostatic hypotension and heart failure, this potential link extends to associations with white matter disease [12, 13]. It is unknown whether these associations represent causality or shared risk factors.

**Fig. 1:**
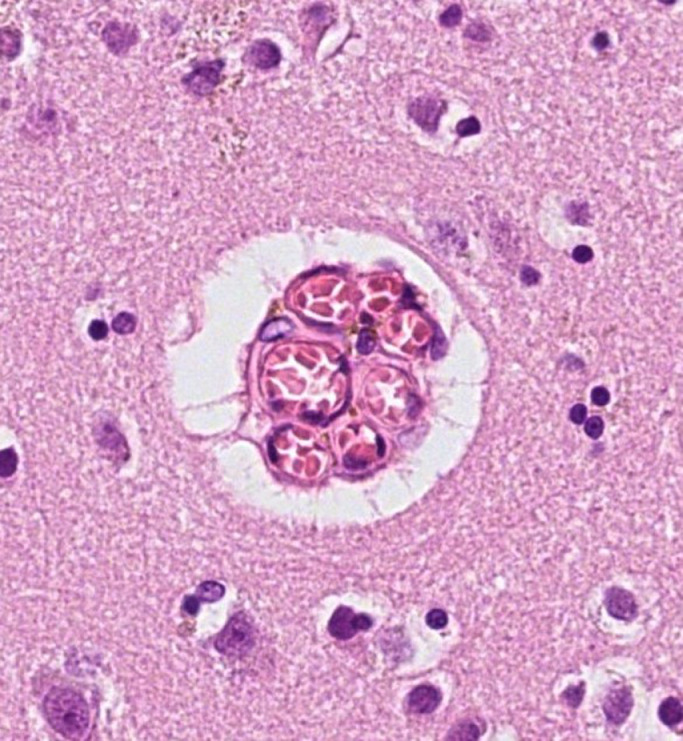
A cortical microvascular ‘raspberry’ in the frontal lobe. Diameter: 45 µm. Stain: haematoxylin and eosin.

In a previous study [14], we examined a microvascular formation of the cerebral cortex, currently termed a ‘raspberry’ due to both simplicity and its appearance under a bright-field microscope (Fig. 1). To our knowledge, the formation has not been previously described but similar findings reported and referred to as ‘vascular bundles’ [15]. We found that raspberries were more common in the cerebral cortex of individuals with neuropathologically verified vascular dementia compared to Alzheimer's disease, frontotemporal lobar degeneration, and non-demented controls. Since ischaemia and the resulting hypoxia is an important pro-angiogenic stimulus [16], we suggested that the higher raspberry density in vascular dementia could indicate angiogenesis induced by cerebral ischaemia. However, raspberries often occurred in seemingly normal cerebral cortex and were unexpectedly frequent in some individuals with only limited ischaemic lesions, including individuals without clinical dementia. We therefore hypothesised that even mild ischaemia, perhaps in the form of transient hypoperfusion, could be sufficient to induce raspberry formation. The aim of the current study was to examine this hypothesis, by quantifying raspberries according to clinical diagnoses and autopsy findings that are believed to be associated with cerebral hypoperfusion. This was an exploratory approach intended to identify potential associations to direct future confirmatory research.

## 2 Materials and methods

### 2.1 Study population

The study population of this retrospective study included adult (age at death ≥ 18 years) individuals who had undergone a diagnostic neuropathological examination at the Department of Pathology, Lund, Sweden, during April 2019–January 2021. Microscopic examination was performed on digitally scanned tissue sections viewed in Sectra IDS7. Data from autopsy reports were accessed through LIMS RS, a digital database where all findings from autopsies performed in Lund are documented. Data from medical records were accessed through the regional medical record system Melior. This is a digital system that includes all public specialist health care in Region Skane, Sweden. Subjects were included if the following criteria were met: a sufficiently extensive neuropathological autopsy (including sections from ≥ 10 different brain regions), access to ≥ 1 section from the frontal lobe, access to data on brain weight, access to data from a complete diagnostic autopsy, and access to clinical data from regional medical records. No specific diagnosis or medical situation was sought; subjects represented consecutively received cases during this 22-month period. All diagnostic work was undertaken prior to this study. The individuals included in this study were not included in our previous study on this topic [14].

### 2.2 Raspberry quantification

**Fig. 2:**
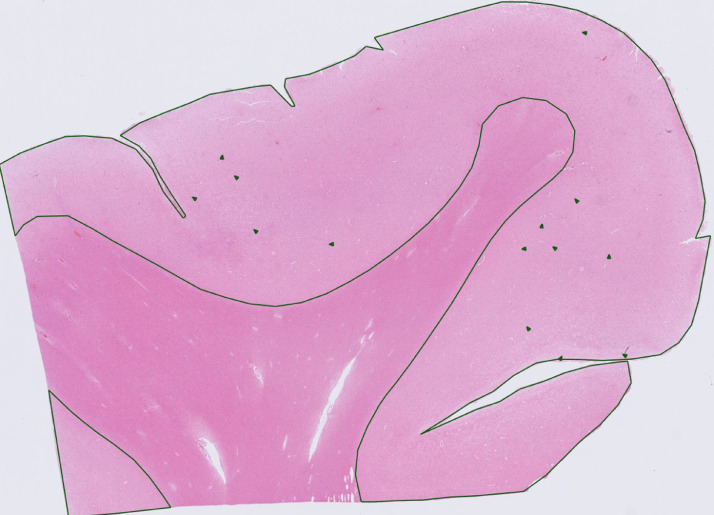
Overview of a tissue section from the anterior frontal lobe. Examined area and observed raspberries have been marked. Stain: haematoxylin and eosin.

For each subject, raspberries were manually counted in the cerebral cortex of a haematoxylin and eosin-stained section of the anterior frontal lobe (Fig. 2). Quantification was performed blinded to data from medical records and autopsy reports. As in our previous study, raspberries were defined as formations composed of ≥ 3 microvascular lumen in immediate proximity to one another, transversally sectioned to simplify identification [14]. The cortical area of the examined section was digitally measured and noted to present raspberry density as raspberries/cm². To adjust for pathology-induced brain weight alterations that could result in incorrect rating of the raspberry density, all raspberry densities were multiplied by the quotient of the individual brain weight divided by previously reported sex-specific mean brain weights [17].

### 2.3 Data from medical records

From the medical records, the following data were collected: hypertension, diabetes mellitus, atrial fibrillation, orthostatic hypotension, chronic heart failure, and acute circulatory failure. Events occurring less than two weeks prior to death were excluded. This decision was based on animal and post-mortem studies demonstrating that a certain minimum of time is required for capillaries to form after an ischaemic event [18, 19]. With this exception, all available data in the medical records was read and considered, as detailed below.

Regarding hypertension, diabetes mellitus and atrial fibrillation, subjects were considered to have these diagnoses if they were documented in the medical records. In the absence of such documentation, subjects were considered free of them. Arterial hypertension was defined as systolic blood pressure ≥ 140 mmHg and/or diastolic blood pressure ≥ 90 mmHg according to European guidelines [20]. No distinction was made based on severity of hypertension, type of diabetes mellitus, or type of atrial fibrillation.

Subjects were considered to have orthostatic hypotension if they presented with typical symptoms in combination with a positive orthostatic test. To assess whether an orthostatic test was positive, criteria from an international consensus definition of orthostatic hypotension were applied [21]. In this consensus definition, criteria are established for orthostatic hypotension, including initial and delayed types. No distinction was made based on type of orthostatic hypotension. Subjects were considered free of orthostatic hypotension if it was undocumented in the medical records. Subjects who had been diagnosed with orthostatic hypotension but had not undergone an orthostatic test were excluded.

The assessment of chronic heart failure was based on echocardiographic findings and – when required – levels of N-terminal pro hormone b-type natriuretic peptide (NT-proBNP). Criteria for echocardiographic findings of heart failure and cut-offs for NT-proBNP were based on European guidelines [22]. No distinction was made based on type or functional classification of heart failure. If heart failure occurred due to an acute event (such as myocardial infarction), subjects were also required to meet the criteria on a clinical follow-up. Subjects were considered free of chronic heart failure if it was undocumented in the medical records. Subjects who had been diagnosed with chronic heart failure (or met some of the criteria), but where the investigation was incomplete, were excluded.

The assessment of acute circulatory failure was based on typical clinical findings, vital parameters, and lab findings described in consensus definitions of shock [23], sepsis, and septic shock [24]. In addition, all subjects who had been diagnosed with cardiac arrest with return of spontaneous circulation or had undergone cardiovascular surgery with perioperative extracorporeal membrane oxygenation were considered to have had acute circulatory failure. Subjects were considered free of acute circulatory failure if it was undocumented in the medical records. Subjects were excluded if there was insufficient data to assess whether an event should be considered acute circulatory failure or not.

### 2.4 Data from autopsy reports

From the autopsy reports, the following data were collected: aortic atherosclerosis, cerebral atherosclerosis, cerebral small vessel disease, cerebral amyloid angiopathy, cerebral infarction, and ischaemic white matter disease. Various significant neuropathological findings were also noted.

The assessment of aortic atherosclerosis has been previously described [25]. Since the prevalence of aortic atherosclerosis was expected to be high, subjects with mild aortic atherosclerosis (scattered atheromatous plaques) were considered free of this pathology. Cerebral atherosclerosis was defined as atherosclerosis of the basal cerebral arteries (the arteries of the circle of Willis and the basilar artery) and included regardless of severity.

Cerebral small vessel disease was defined as small vessel atherosclerosis, lipohyalinosis, and arteriolosclerosis [26]. Cerebral amyloid angiopathy was counted as a separate variable due to its distinct pathology and association with neurodegenerative disease [27].

No distinction was made based on type of cerebral infarction. Ischaemic white matter disease was defined as a reduction of oligodendrocytes and myelin, mild reactive gliosis, and accompanying stenosing small vessel disease (but not necessarily focal infarcts), giving an overall impression of ischaemic damage [28]. Subjects were considered free of this pathology if it was absent or considered non-ischaemic (secondary to neurodegeneration) [28]. Subjects diagnosed with white matter disease where a convincing or presumable aetiology could not be established, were excluded.

### 2.5 Statistics

Statistical analyses were undertaken in IBM SPSS Statistics Version 25. The outcome variable – raspberry density – was deemed sufficiently normally distributed to allow parametric testing. Independent-samples *t*-test was used to compare the mean raspberry density between groups defined by the categorical (binary) exposure variables under study. To analyse the association between raspberry density and age, simple linear regression was used. Following these tests, multiple linear regression was used to analyse the association between raspberry density and hypertension, diabetes mellitus, acute circulatory failure, cerebral atherosclerosis, and ischaemic white matter disease (number of variables limited due to sample size). Prior to inclusion in multiple linear regression, exposure variables were tested in order not to miss strong intervariable correlations that could otherwise hide weak exposure-outcome associations (Pearson correlation).

A *p value* of ≤ 0.05 was considered statistically significant. Effect sizes were measured by 95% confidence intervals (95% CI). Continuous variables were presented as mean (standard deviation) and categorical variables as frequency (percentage), unless otherwise indicated.

### 2.6 Ethical approval

The study was approved by the Swedish Ethical Review Authorities, applications number 1143-2018 and 00080-2019.

## 3 Results

**Fig. 3:**
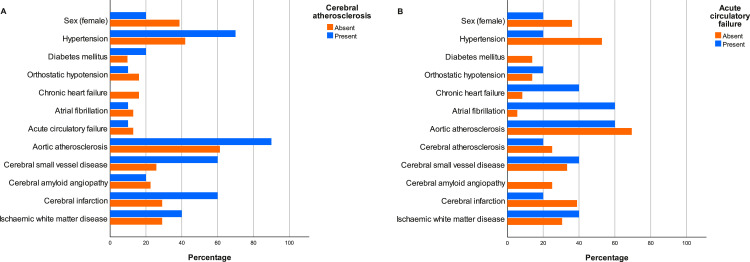
(A) Percentages of background variables and exposure variables according to presence (blue bars) or absence (orange bars) of cerebral atherosclerosis. Presence of cerebral atherosclerosis: *n* = 15, mean age = 75.9 (6.5) years. Absence of cerebral atherosclerosis: *n* = 44, mean age = 70.4 (10.9) years. (B) Percentages of background variables and exposure variables according to presence (blue bars) or absence (orange bars) of acute circulatory failure. Presence of acute circulatory failure: *n* = 10, mean age = 71.7 (15.5) years. Absence of acute circulatory failure: *n* = 48, mean age = 71.1 (8.4) years.

Of 106 subjects, 62 met the inclusion criteria. The mean age was 71.9 (10.0) years, ranging from 46–97 years. 21 (33.9%) of the 62 subjects were female. Regarding clinical features, 33 subjects (53.2%) had hypertension and 10 (16.1%) had diabetes mellitus. Of the 10 subjects with acute circulatory failure, 3 cases were caused by cardiac arrest with return of spontaneous circulation, 2 by cardiac surgery, 3 by cardiogenic shock, and 2 by sepsis. Time from acute circulatory failure to death varied from 3 weeks to > 10 years. Frequencies of background variables and exposure variables are presented in Table 1. Due to the observed differences in raspberry density (see below), percentages of background variables and exposure variables according to cerebral atherosclerosis and acute circulatory failure are presented in Fig. 3.

**Table 1:** Frequencies and percentages of background variables and exposure variables. For each row, 'Present/Absent' shows the number of subjects who have the specified variable, followed by the number of subjects who do not. 'Percentage' shows the fraction of subjects who have the specified variable. Some subjects were excluded due to missing or insufficient data. SD = standard deviation.

Background data			
**Variable**	**Mean**	**SD (range)**	**Included/Excluded**
Age (years)	71.9	10.0 (46-97)	62/0
**Variable**	**Female/Male**	**Percentage (female)**	**Included/Excluded**
Sex	21/41	33.9	62/0

**Clinical data**			

**Variable**	**Present/Absent**	**Percentage**	**Included/Excluded**
Hypertension	33/29	53.2	62/0
Diabetes mellitus	10/52	16.1	62/0
Orthostatic hypotension	8/51	13.6	59/3
Chronic heart failure	7/44	13.7	51/11
Atrial fibrillation	17/45	27.4	62/0
Acute circulatory failure	10/48	17.2	58/4

**Pathological data**			

**Variable**	**Present/Absent**	**Percentage**	**Included/Excluded**
Aortic atherosclerosis	45/17	72.6	62/0
Cerebral atherosclerosis	15/44	25.4	59/3
Cerebral small vessel disease	24/38	38.7	62/0
Cerebral amyloid angiopathy	14/48	22.6	62/0
Cerebral infarction	27/35	43.5	62/0
Ischaemic white matter disease	21/38	35.6	59/3

**Fig. 4:**
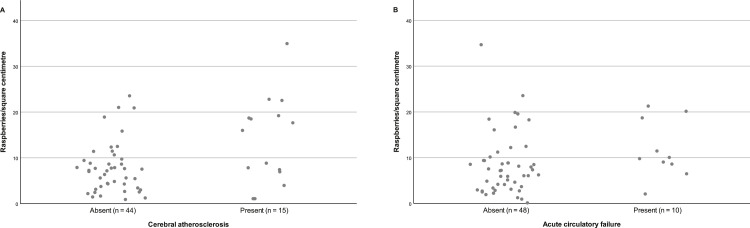
Cortical raspberry density of individual subjects according to presence or absence of (A) cerebral atherosclerosis, and (B) acute circulatory failure.

The mean examined cortical area was 1.73 (0.45) cm². The overall mean raspberry density was 8.9 (6.8) raspberries/cm², ranging from 1.4 raspberries/cm² to 35.7 raspberries/cm². The largest observable difference was a higher raspberry density in subjects with cerebral atherosclerosis (13.6 [9.5] raspberries/cm² vs. 7.4 [5.1] raspberries/cm²), followed by acute circulatory failure (11.8 [6.9] raspberries/cm² vs. 8.0 [6.6] raspberries/cm²) and ischaemic white matter disease (10.8 [8.0] raspberries/cm² vs. 8.1 [6.2] raspberries/cm²). The higher raspberry density in subjects with cerebral atherosclerosis was statistically significant (*p =* 0.029 [95% CI 0.7, 11.6]), whereas the other differences were not (*p =* 0.12 [95% CI -0.9, 8.4] and *p =* 0.16 [95% CI -1.1, 6.4] for acute circulatory failure and ischaemic white matter disease, respectively). Raspberry density increased slightly with age (β 0.15), but not statistically significantly so (*p =* 0.092 [95% CI -0.03, 0.32]). The raspberry density was also slightly higher in females, subjects with hypertension, atrial fibrillation, and cerebral small vessel disease. It was slightly lower in subjects with chronic heart failure and cerebral amyloid angiopathy, and similar between subjects with and without diabetes mellitus, aortic atherosclerosis, and cerebral infarcts. These differences were not statistically significant. Raspberry density according to all background variables and exposure variables is presented in Table 2. A complete distribution of raspberry density according to cerebral atherosclerosis and acute circulatory failure is presented in Fig. 4.

**Table 2:** Mean cortical raspberries/cm² according to background variables and exposure variables. For each row, subjects have been grouped based on presence or absence of the specified variable and their raspberry densities presented. Effect sizes and *p value*s are based on simple linear regression for age and independent-samples *t*-test for remaining variables. SD = standard deviation. CI = confidence interval.

Background data				
**Variable**	**n/a**	**n/a**	**Effect size (95% CI)**	***p value***
Age	n/a	n/a	β=0.15 (-0.03, 0.32)	0.092
**Variable**	**Female (mean (SD))**	**Male (mean (SD))**	**Effect size (95% CI)**	*p value*
Sex	10.4 (8.0)	8.1 (6.1)	2.2 (-1.4, 5.9)	0.22

**Clinical data**				

**Variable**	**Present (mean (SD))**	**Absent (mean (SD))**	**Effect size (95% CI)**	*p value*
Hypertension	9.8 (7.5)	7.9 (5.9)	1.9 (-1.6, 5.4)	0.28
Diabetes mellitus	8.7 (7.6)	9.0 (6.7)	-0.3 (-5.0, 4.4)	0.90
Orthostatic hypotension	9.5 (6.1)	9.1 (7.1)	0.4 (-4.9, 5.8)	0.87
Chronic heart failure	7.4 (1.9)	8.7 (7.2)	-1.3 (-6.8, 4.2)	0.32
Atrial fibrillation	10.2 (7.1)	8.4 (6.8)	1.8 (-2.1, 5.6)	0.37
Acute circulatory failure	11.8 (6.9)	8.0 (6.6)	3.7 (-0.9, 8.4)	0.12

**Pathological data**				

**Variable**	**Present (mean (SD))**	**Absent (mean (SD))**	**Effect size (95% CI)**	*p value*
Aortic atherosclerosis	8.8 (7.2)	9.1 (5.8)	-0.3 (-4.2, 3.6)	0.87
Cerebral atherosclerosis	13.6 (9.5)	7.4 (5.1)	6.1 (0.7, 11.6)	0.029
Cerebral small vessel disease	10.0 (7.9)	8.1 (6.1)	1.9 (-1.7, 5.4)	0.29
Cerebral amyloid angiopathy	7.4 (6.3)	9.3 (7.0)	-1.9 (-6.1, 2.2)	0.35
Cerebral infarction	9.3 (7.5)	8.6 (6.4)	0.7 (-2.8, 4.2)	0.69
Ischaemic white matter disease	10.8 (8.0)	8.1 (6.2)	2.7 (-1.1, 6.4)	0.16

The positive association between raspberry density and cerebral atherosclerosis remained statistically significant (*p =* 0.003 [95% CI 2.3, 11.1]) in multiple linear regression where hypertension, diabetes mellitus, acute circulatory failure, and ischaemic white matter disease were also included (Table 3). No other associations reached statistical significance. Included exposure variables did not correlate strongly, or at a statistically significant level (Pearson correlation, data not shown).

**Table 3:** Mean cortical raspberries/cm² according to exposure variables selected for multiple linear regression. For each row, subjects have been grouped based on presence or absence of the specified variable and their raspberry densities presented. Effect sizes and *p value*s are based on multiple linear regression (see Table 2 for univariable analyses). SD = standard deviation. CI = confidence interval.

Variable	Present (mean (SD))	Absent (mean (SD))	Effect size (95% CI)	*p value*
Hypertension	9.8 (7.5)	7.9 (5.9)	1.1 (-2.8, 4.9)	0.58
Diabetes mellitus	8.7 (7.6)	9.0 (6.7)	-3.5 (-9.3, 2.2)	0.22
Acute circulatory failure	11.8 (6.9)	8.0 (6.6)	3.4 (-1.45, 8.2)	0.17
Cerebral atherosclerosis	13.6 (9.5)	7.4 (5.1)	6.7 (2.3, 11.1)	0.003
Ischaemic white matter disease	10.8 (8.0)	8.1 (6.2)	-0.6 (-4.7, 3.5)	0.77

**Fig. 5:**
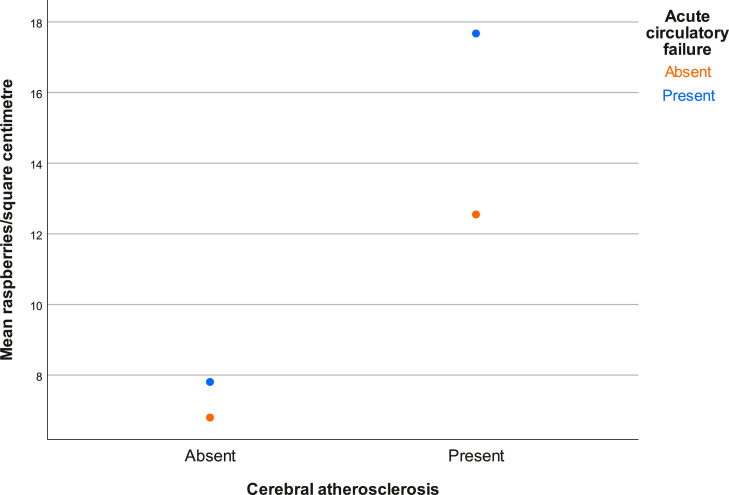
Mean cortical raspberry density according to cerebral atherosclerosis and acute circulatory failure. Neither condition: *n* = 35. Only cerebral atherosclerosis: *n* = 10. Only acute circulatory failure: *n* = 6. Both conditions: *n* = 4.

Raspberry density differed more between subjects with and without acute circulatory failure if subjects also had cerebral atherosclerosis. Further, subjects with both cerebral atherosclerosis and acute circulatory failure had higher raspberry density than subjects with only one or none of these conditions. Due to the limited number of subjects in a number of the categories, no statistical analysis was performed on this data. It is presented graphically in Fig. 5.

## 4 Discussion

### 4.1 Raspberries and hypoperfusion

The most prominent finding of this study on cortical microvascular raspberries is the higher raspberry density in subjects with cerebral atherosclerosis. No other variable indicated a variation in raspberry density of similar magnitude, or of statistical significance. Further, the association remained in (limited) multiple linear regression. The contrasting lack of association between raspberry density and aortic atherosclerosis is also an interesting finding. However, our wide confidence interval indicates that we are unsure of the size and relevance of this association. Consequently, the results would benefit from replication in an independent (preferably larger) study sample.

Large vessel disease has been associated with cerebral hypoperfusion [29]. This has generally been in the form of severe arterial stenosis, while our study included atherosclerosis regardless of severity. One study has shown an association between asymptomatic cerebral atherosclerosis and reduced cerebral blood flow but suggested this was due to shared risk factors [7]. Likewise, a plausible explanation is that raspberry formation and cerebral atherosclerosis are associated by shared risk factors. If there is instead a causal link between cerebral atherosclerosis and raspberry formation, it might be that of disrupted cerebral autoregulation. While neurovascular coupling of small blood vessels regulates local perfusion based on neuronal activity [30], it is possible that larger arteries contribute to autoregulation aimed at maintaining blood flow despite variations in blood pressure [31]. The stiffer atherosclerotic arteries might be unfit for autoregulation and make the brain more susceptible to hypoperfusion caused by episodic hypotension. Cerebral blood flow would remain unaffected under normotension, resulting in temporary rather than chronic hypoperfusion.

The hypothetical link to episodic hypotension leads us to our finding of a higher cortical raspberry density in subjects with acute circulatory failure. The difference in raspberry density between subjects with and without this condition was the second largest observable in the study. While not statistically significant, type II error is difficult to rule out. Acute circulatory failure as defined in this study has been associated with cerebral hypoperfusion and resulting ischaemia [1], [2], [3], [4]. The consequences include damage to the cerebral cortex, indicating this as a potential location for pro-angiogenic stimulation. Time between acute circulatory failure and death varied among our subjects. Since adult cerebral vasculature is believed to be independent of pro-angiogenic stimulation [32], it is possible that once formed, the blood vessels remain even after the triggering stimulus has ceased. In our study, acute circulatory failure was broadly defined and the study sample small. It is necessary to further assess whether there is an association between cortical raspberries and acute hypoperfusion. This would require a larger study sample with variables that are easier to objectively define, such as cardiac arrest with return of spontaneous circulation alone. This would also allow for examinations of raspberry density according to survival time and immunohistochemical assessment of pro-angiogenic factors.

It has been suggested that acute hypotension combined with significant arterial stenosis makes the brain particularly susceptible to hypoperfusion [33]. It has also been suggested that hypoperfusion itself can disable cerebral autoregulation and thus further worsen the ischaemia [4]. If the normal autoregulatory function of cerebral arteries has already been compromised from atherosclerotic stiffening, the combination of cerebral atherosclerosis and acute circulatory failure may be harmful even in the absence of significant stenosis. Since our descriptive data indicated a particularly high cortical raspberry density in the small number of subjects who suffered from this combined pathology, the currently unanswered question of whether such an interaction occurs should be addressed in future studies.

The part of our study that examined cortical raspberries according to pathological markers of chronic or recurrent hypoperfusion (ischaemic white matter disease and cerebral small vessel disease) was inconclusive. These pathologies are strongly associated, with hypoperfusion as a possible link between them [5]. They are also considered important substrates for vascular dementia, where we found a higher, statistically significant raspberry density in our previous study [14]. The (often) severe nature of the ischaemic damage that is associated with vascular dementia could explain the discrepancy between the results of our previous and current work. One approach to further address this topic would be to examine raspberry density according to quantified cerebral small vessel disease and severity-graded ischaemic white matter disease.

Differences in cortical raspberry density according to our clinical markers of chronic or recurrent hypoperfusion (orthostatic hypotension, chronic heart failure, and atrial fibrillation) were inconclusive, minor, and divergent. These conditions have previously been associated with reduced cerebral blood flow [8], [9], [10], [11]. In one study, an association between orthostatic hypotension and reduced cerebral blood flow during supine position was identified [34]. However, in larger studies of orthostatic hypotension (and atrial fibrillation), cerebral blood flow did not differ from controls when subjects were free of ongoing orthostatic symptoms or arrhythmia [11, 35]. Similarly, reductions of cerebral blood flow and tissue oxygen saturation in subjects with chronic heart failure were more pronounced during upright position [36, 37], indicating temporal variation in supposed hypoperfusion. We found no indications that such (likely temporary) reductions in cerebral blood flow would be severe enough to induce raspberry formation in the cerebral cortex. These results have limitations. Subjects with orthostatic hypotension and heart failure were few. Several subjects were excluded during the assessment of heart failure due to insufficient data in the medical records (although the prevalence of heart failure among included subjects could still be representative of that in an older population [38]). Our material was too small to form subgroups based on these variables, such as functional classification of heart failure, type of atrial fibrillation, or presence/absence of compensatory increase in heart rate in orthostatic hypotension. Consequently, the question of whether chronic or recurrent hypoperfusion could be associated with raspberry formation remains unanswered.

Finally, it cannot be negated that cortical raspberry formation depends partially on age. However, the effect would likely be small and could be driven by an accumulation of risk factors rather than a true effect of aging. The prevalence of diabetes mellitus and hypertension were similar to that of the general population [39, 40].

### 4.2 Strengths and limitations

The exploratory design of this study was motivated by the limited previous research on raspberries. The goal of this study was to direct future research, rather than providing definite conclusions. When deemed appropriate, variables were defined based on reproducible criteria rather than the presence of a diagnosis in the medical records alone. A number of limitations have already been mentioned. In addition to these, the study was limited by its retrospective, observational design. The estimated raspberry density was based on a limited area of the cerebral cortex (the anterior frontal lobe). This made the measurement less precise but had an equal probability of affecting all subjects. Finally, while having a broad approach from a haemodynamic perspective, other mechanisms that could potentially induce raspberry formation, such as euvolemic anaemia and respiratory failure, were not covered.

### 4.3 Conclusion

This exploratory study indicates that cortical raspberries could be associated with cerebral atherosclerosis. The remaining results were inconclusive but motivate further examination of variables such as acute circulatory failure. A confirmatory approach is required to examine these potential associations before conclusions can be drawn.

## Funding

This work was supported by the Trolle-Wachtmeister Foundation for Medical Research and Region Skane.

## Author contributions

Henric Ek Olofsson collected and analysed the data and drafted the manuscript. All authors conceived the study and reviewed and edited the manuscript.
